# Remarks on Inflammation and Its Curability

**Published:** 1876-06

**Authors:** E. F. Starr

**Affiliations:** Nacoochee, Ga.


ATLANTA

MEDICAL AND SURGICAL JOURNAL

VOL. XIV.] JUNE—1876. [No. 3

### Original Communications.

### REMARKS ON INFLAMMATION AND ITS CURABILITY.

BY E. F. STARR, M.D., OF NACOOCHEE, GA.

The skillful and efficient treatment of the acute inflammatory affections is one of the most important duties of the physician. The usual suddenness and violence of their onset, the rapidity of their course, and their strong tendency to fatality, render the demand for skill and efficiency urgent and imperative.

Without attempting to define the term, we concur in opinion with Dr. Dugas (see ATLANTA MEDICAL AND SURGICAL JOURNAL, for December,) that the different forms of inflammation should be regarded as so many entities and treated as such. "They differ in causation, in symptoms, in duration, in terminations and in curability," and it may be added, in requirements of treatment. This being the case, the fact (if it be a fact) that bleeding will not "cure a mere pimple on the face," is neither evidence nor proof that it will not cure some forms of inflammation; for it seems that extensive and grave inflammations, that affect the general circulation, are more amenable to general treatment than those of a more circumscribed and trivial character.

Probably Broussais' greatest error was the confounding of all the forms of inflammation into one, and contending that the ulcerative and other diseased processes occurring in the course of essential fevers were amenable to the same treatment as the ordinary phlegmasiæ; and the fact that all grades and qualities of inflammation are not alike, nor equally curable by the same treatment, has probably had much to do with the prevailing tendency in the profession to regard blood-letting as unnecessary in any. This conclusion, however, upon this basis, is certainly unphilosophical and untenable.

Dr. Dugas, after having long taught that inflammation was not curable, became convinced to the contrary—at least in regard to some forms of it, by having positive ocular demonstration of cure. Now if some forms of inflammation are shown to be curable, may we not hope to find that other forms are also curable? Now that the investment of unbelief is penetrated, may we not hope to enucleate the whole matter? At all events the inability theory is not likely to promote or give impetus to medical progress. But let us agree what it is to cure inflammation. Is it not to arrest its progress in a reasonably short time, and cause it to terminate in resolution, when if left alone it would not act so benignly?

If this is what we are to understand, might not other instances be cited of cures as unequivocal as those of Dr. Dugas? What have Dr. Campbell and others said in reference to the arrest of inflammation in the extremities by the ligation of arteries supplying the inflamed parts? Do they not claim to have obtained important results by so doing? This class of cases, too, come under ocular observation. Upon what principle are these results obtained? Is it not that of diminishing the amount of blood allowed to flow to the parts affected? In this class of cases the effect is direct. In the other, of feruncle and erysipelas cured by external application, it is probably indirect, through the vaso-motor nervous influence. Suppose, however, the disease to be seated in an internal vital organ, how is destructive lesion to be prevented? Is it not upon the same principle, that of protecting the suffering organ from the rush of heated blood, with its surcharge of infiltrating and indurating materials, tending to final stasis? But how can this protection be given? Can an artery be tied for this purpose? Can the kidneys, the peritoneum, the lungs, the brain, etc., be protected by the tying of arteries? Nay, verily! but a vein may be corded and opened, and thus efficient protection given; and the nervous batteries and the heart may be bridled by veratrum and gelsiminum, and quiet restored. Then resolution comes and health returns.

CASE.—Mr. B., æt. thirty, was attacked with acute nephritis. He had great suffering, pain and tenderness in the region of the kidneys, raging fever, and disturbed urinary function—the secretion at first increased and then suppressed. I saw him in the forenoon, but he had been attacked on the previous day. The case was urgent; there was no time for delay, and I acted promptly. I corded a vein and drew about a quart of blood. This afforded him some relief, but I waited with him until the afternoon and repeated the bleeding to about the same extent. This gave him so much relief that he had rest for the night; the urine resumed its flow. On the following day I repeated the blood-letting, after which there was no further trouble; convalescence commenced, and he had a rapid and complete recovery.

Some auxiliaries were used, but the chief reliance was upon the lancet. Was this case cured? But I would not bleed this way in every case; circumstances must determine. Again, veratrum may be made to overcome some cases of inflammation as promptly and as completely as can be reasonably desired. Again and again have I used it for this purpose with prompt and happy effect, and I can assert that under the combined and skilful use of the lancet and veratrum, common sthenic inflammation cannot progress, nor can it long exist.

In the treatment of inflammation of the vital organs, time—the saving or improvement of time by prompt effect, by early, sharp, decisive treatment—is of the utmost importance. The cautious, do-nothing, expectant method will not do in this class of cases. Many patients have been lost by frittering away the precious hours in the use of temporizing, palliative, or uncertain remedies and expedients. Through fear of debility, many cases of inflammation have been allowed to run rapidly into fatal lesion, and the patients consequently into the debility and stasis of death. In my younger days I had a patient (J. H.), a well-grown lad of twelve or fourteen years. He was seized with sharp, agonizing pains in the lower bowels, spreading up, with characteristic fever and pulse. After trying some palliatives, warm bath, etc., for a little while without relief, I decided I had a case of peritonitis. I resorted to the lancet, and bled freely once. This afforded the patient comparative ease and a respite of some duration from acute suffering. The nature of the case, however, being serious, a consultation was agreed upon. (A cathartic had previously been administered by the family, which acted on the bowels.) Dr. —— was called—an older and more distinguished medical man. I suggested the propriety and necessity of repeating the bleeding, in order to keep up the impression made by the first (which had acted so happily), and to prevent too early a reaction and rekindling of the inflammation. He thought it would hardly be necessary, but preferred rather that a dose of oil should be given, to carry off crude substances that he supposed might be in the bowels. The cathartic was given; the sufferings returned; the inflammation rekindled; further bleeding was not practiced; the debility of disease and fatal lesion ensued, and the patient died. I was impressed by the result of this case with the importance of maintaining the good impression early made in such cases, and I would prefer to bleed a patient ten times, if need be, rather than let him die.

In other cases of peritonitis, where I have not stinted the lancet, I have been successful. If I can prevent a general peritonitis from terminating fatally, I consider that I *cure* the inflammation. Since the introduction of veratrum by Dr. Norwood, as a general rule inflammation may be treated (and I think cured) with less bleeding than formerly. But beware; do not sleep through the precious hours while the case is savable; do not throw away the opportunity offered in the early stage. My experience has made me a strong believer in the crushing-out process for these terrible onsets of acute inflammation, and when I see such as usually terminate fatally if left alone or mistreated, "hide their diminished heads" and speedily retire under the vigorous application of the lancet, veratrum, etc., I cannot disbelieve my senses, nor ignore my experience, nor refrain from the conclusion that they have been cured.

